# Salmonella Weltevreden lung abscess and empyema without preceding gastrointestinal symptoms: an emerging pathogen in Australia?

**DOI:** 10.1099/acmi.0.000635.v3

**Published:** 2024-10-30

**Authors:** Victoria Grey, Ernest Tee, Lauren Phillips, Gino Micalizzi, Mark Armstrong

**Affiliations:** 1 Infection Management Services, Logan Hospital, Meadowbrook, Queensland, Australia; 2 School of Medicine, University of Queensland, Brisbane, Queensland, Australia; 3 Infectious Diseases Department, Nepean Hospital, Kingswood, New South Wales, Australia; 4 Infection Management Services, Mater Hospital, Brisbane, Queensland, Australia; 5 Public Health Microbiology, Queensland Public Health and Scientific Services, Coopers Plains, Australia

**Keywords:** empyema, immunocompetent, Queensland, *Salmonella*

## Abstract

Non-typhoidal *Salmonella* lung infections are rare and are usually confined to immunocompromised hosts. Previous case reports have found that usually patients have either gastroenteritis or bacteraemia in addition to pulmonary involvement. We present the first known reported case of a *Salmonella* Weltevreden lung abscess and empyema in an immunocompetent patient without gastroenteritis. Despite the use of antimicrobials active against the pathogen, the patient needed surgical intervention to achieve adequate source control. While *S*. Weltevreden has previously been associated with returned travellers, especially from Southeast Asia, its incidence in Queensland is now increasing. Therefore, it is important for clinicians to be aware of its potential severity as well as the range of presentations.

## Data Summary

No data were generated during this research, nor is it required for the work to be reproduced.

## Introduction

*Salmonella* belongs to the Enterobacteriaceae family and is a Gram-negative, motile and facultative anaerobic organism [1]. *Salmonella* can be divided into two species: *Salmonella enterica* and *Salmonella bongori* (which is rarely pathogenic in humans) [1]. *S. enterica* can then be further divided into six subspecies. Members of each species can then be serotyped according to their surface antigens using the White–Kauffmann–Le Minor scheme [1,2].

In Queensland, salmonellosis cases are notifiable to the Department of Health, and an isolate is referred to the Public Health Microbiology, *Salmonella* Reference Laboratory for typing. To date, *S*. Weltevreden is one of over 2600 serovars of *Salmonella* spp. that have been recognized and classified for the epidemiological surveillance of salmonellosis [2].

*S. enterica* serovar Weltevreden has emerged in the last 20 years as a common food-borne pathogen in Southeast Asia [3]. It most commonly causes gastroenteritis, with more invasive disease reported rarely and usually in immunocompromised hosts. To our knowledge, this is the first case of *S*. Weltevreden empyema with an associated lung abscess, and it occurred in an immunocompetent patient without any associated gastrointestinal symptoms.

## Case report

**Fig. 1.**
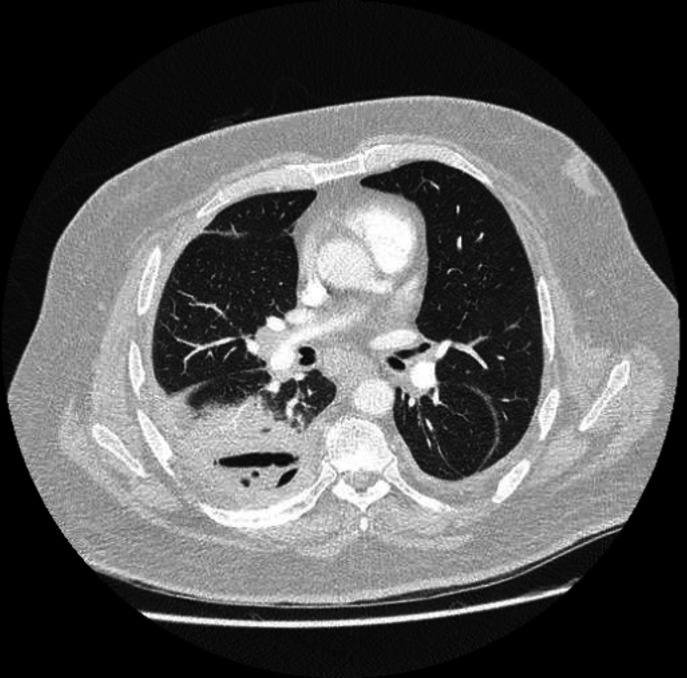
Axial CT image of the chest demonstrating right-sided lung abscess and empyema.

A 50-year-old Aboriginal male presented with a 3-week history of fever, fatigue and productive cough to a Queensland hospital in 2022. The patient was an active smoker with a history of poorly controlled diabetes mellitus type 2 and ischaemic heart disease. The patient had no recent travel history. The patient had no diarrhoea or other gastrointestinal symptoms. His initial chest X-ray showed a loculated moderate right-sided pleural effusion. The patient received several different antibiotics, including intravenous piperacillin–tazobactam, ceftriaxone, meropenem and azithromycin but continued to deteriorate with persistent fever, hypoxia and rising inflammatory markers. A repeat CT of the chest showed persisting empyema with an adjacent right lower zone lung abscess, as demonstrated in Fig. 1. Multiple blood cultures (however only one set was taken prior to the administration of intravenous antibiotics) remained negative. Eight days after the admission, a pleural fluid aspirate demonstrated moderate Gram-negative bacilli (GNB), and one colony of *S*. Weltevreden was cultured. Pleural fluid showed leucocytes of 396×10^6^/L (97% mononuclear). The significance of the isolate growth was initially questioned, with the possibility of contamination raised as one colony of this organism in this site was thought to be unusual and out of keeping with the many GNB seen in the Gram stain of the sample. Further culture plates along with a nutrient broth were re-set up from the sample but failed to grow the organism again. 16S rRNA sequencing as well as *Salmonella* PCR were requested on the original pleural fluid sample, and this confirmed the presence of the organism, making it unlikely to be a contaminant. Culture and *Salmonella* PCR were not performed on a stool specimen, as he never had gastrointestinal symptoms. Due to the failing medical management of his empyema, the patient underwent video-assisted thoracoscopic surgery (VATS) decortication on day 21 of the admission. The culture at this time was negative; however, the histology was consistent with an empyema. The patient improved clinically after this procedure and was discharged 2 days later. The patient remained well after completing 4 weeks of oral amoxicillin post-surgery.

## Discussion

Lung infections due to non-typhoidal *Salmonella* are uncommon, especially in the absence of immunosuppression [4]. Most case reports to date have been due to *S*. Typhimurium, *S*. Choleraesuis or *S*. Enteritidis [5,7]. A 2005 case series of 12 patients with non-typhoidal *Salmonella* empyema found that *S*. Choleraesuis followed by *S*. Typhimurium were responsible for the majority of cases, with ten of these cases having significant underlying immunosuppression. More invasive diseases and higher mortality rates were observed among the *S*. Choleraesuis group, with many of the patients having positive cultures from multiple sites [7]. A higher mortality rate was observed despite the higher rates of appropriate antimicrobial therapy, suggesting that more aggressive surgical management is potentially required for the management of *S*. Choleraesuis [7]. *Salmonella* serotypes causing empyema have been described as carrying virulence plasmids, which may account for different presentations and invasiveness [8]. A study by Zhang *et al*. performed whole-genome sequencing on 96 *S*. Weltevreden isolates in China [9]. While most isolates did not harbour antimicrobial resistance genes, many did carry an IncFII(S) type plasmid, which was thought to contribute to enhanced bacterial pathogenesis [9]. Despite the predominance of empyema case reports being in immunocompromised patients, a recent review has identified six reported cases of *S*. Enteritidis pulmonary infection in immunocompetent patients, and the majority were associated with gastroenteritis on presentation [4].

To our knowledge, our case represents the first reported case of a lung abscess and empyema caused by *S*. Weltevreden. In contrast to many other reported non-typhoidal *Salmonella* lung infections, this case was not associated with gastroenteritis or bacteraemia. *S*. Weltevreden is an emerging pathogen with outbreaks in human beings associated with the consumption of animal-source foods [10,11]. It has been isolated in Asian seafood, geckos, pigs and poultry [12,14]. Although *S*. Weltevreden infections primarily cause self-limiting acute gastroenteritis, rare extra-intestinal manifestations of *S*. Weltevreden have been reported, ranging from soft tissue infections to calcaneal osteomyelitis and bacteraemia [5,15]. Fatal cases due to severe sepsis have also been reported, with risk factors for invasive S. Weltevreden infections including malignancy and iatrogenic immunosuppression [16]. Our case had no history of either but did have type 2 diabetes mellitus, which has also been described as a potential risk factor for * S*. Weltevreden infection [16].

**Fig. 2.**
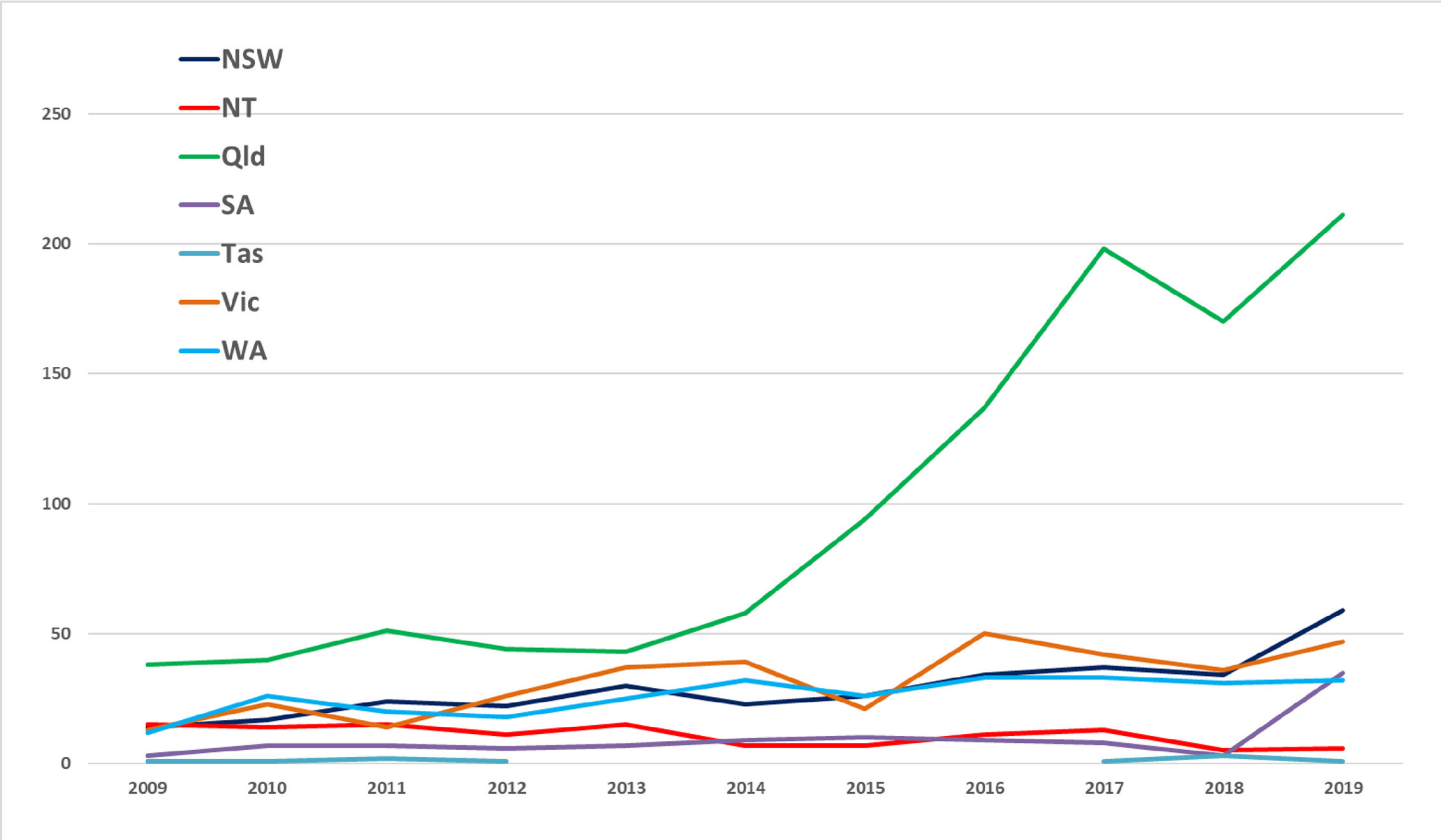
Human cases of *S*. Weltevreden observed in Australia from 2005 to 2019 [18]. Abbreviations: NSW, New South Wales; NT, Northern Territory; Qld, Queensland; SA, South Australia; Tas, Tasmania; Vic, Victoria; WA, Western Australia.

Historically, cases of *S*. Weltevreden in Queensland have been associated with individuals returning from overseas, particularly from Southeast Asia [17]. However, the percentage of all human salmonellosis cases attributed to *S*. Weltevreden in Queensland has increased from 1.6% in 2009 to 5.6% in 2019 [18]. In 2019, a nationwide outbreak of *S*. Weltevreden associated with packaged frozen meals occurred: over 50 individuals were unwell with a diarrhoeal illness, prompting a media release and recall of the implicated food products [19]. No further large outbreaks have been reported, but despite this, *S*. Weltevreden is now considered endemic within Australia, particularly within Queensland, where it is commonly isolated from environmental sources [17]. Wild Asian house geckos introduced from Southeast Asia are thought to be one of the main reservoirs responsible for the transmission of the disease [17]. Fig. 2 shows the trend of human cases of *S*. Weltevreden observed in Australia from 2005 to 2019 and highlights that significantly more are being observed in Queensland now compared to other states.

The isolate, in our case, remained sensitive to amoxicillin, ceftriaxone, ciprofloxacin and co-trimoxazole. Out of the 46 Australian isolates, between 1995 and 2001, only 2.2% were resistant to ampicillin, streptomycin, sulfamethoxazole, tetracycline and trimethoprim [20]. The antimicrobial resistance genetic elements in *S*. Weltevreden are shared with those in other Enterobacteriaceae, with well-described resistance genes such as *bla*_TEM_ (conferring resistance to ampicillin), *sul1* and *sul2* (conferring resistance to sulfamethoxazole) and various *tet* genes (conferring resistance to doxycycline) sequenced [20].

## Conclusion

This case is important as it highlights that lung abscess and empyema are other potential manifestations of *S*. Weltevreden. VATS-decortication formed a vital part of this patient’s treatment in conjunction with directed antibiotic therapy once the causative pathogen was known. Given the absence of any travel history or gastroenteritis and the unusual focus of infection, the microbiological diagnosis of *S*. Weltevreden in this patient was initially questioned. It is likely that the single colony of *S*. Weltevreden grew as a result of the extensive antibiotic treatment active against the organism prior to obtaining pleural fluid. The discordant Gram stain appearance was likely due to many non-viable bacteria. This highlights the benefit of obtaining samples early, ideally before antibiotic therapy is administered, to help increase diagnostic yield as well as not disregarding positive cultures with only limited growth. The incidence of *S*. Weltevreden is increasing in Queensland, and it is important for clinicians to be aware of its pathogenicity and a wide variety of clinical presentations, including in immunocompetent hosts.

## Abbreviations

GNB: gram-negative bacilli
VATS: video-assisted thoracoscopic surgery
